# Vascular changes and their implications in lipedema

**DOI:** 10.3389/fcell.2026.1819443

**Published:** 2026-05-08

**Authors:** Sara Al-Ghadban, Yuqi Guo, Zuzanna J. Juskiewicz, Rachelle Crescenzi, Xin Li, Brant E. Isakson, Scott T. Hollenbeck

**Affiliations:** 1 Department of Plastic Surgery, Maxillofacial and Oral Health, University of Virginia, Charlottesville, VA, United States; 2 Robert M. Berne Cardiovascular Research Center, University of Virginia School of Medicine, Charlottesville, VA, United States; 3 Department of Radiology and Medical Imaging, University of Virginia, Charlottesville, VA, United States; 4 UVA Comprehensive Cancer Center, University of Virginia School of Medicine, Charlottesville, VA, United States; 5 Department of Molecular Physiology and Biophysics, University of Virginia School of Medicine, Charlottesville, VA, United States

**Keywords:** adipose tissue, extracellular matrix (ECM), Lipedema, lymphatic vessels, vascular dysfunction

## Abstract

Lipedema is a chronic, multifactorial disorder characterized by connective tissue dysregulation, in which vascular dysfunction plays a significant role. Lipedema manifests as symmetrical, painful accumulation of adipose tissue, predominantly in the lower body and arms, with progressive pain, tissue heaviness, and soft-tissue changes across disease stages. Emerging evidence from the micro-to macro-scale implicates endothelial dysfunction, aberrant angiogenesis, and vessel fragility in the pathological accumulation of interstitial fluid leading to tissue edema. Vascular changes are compounded with extracellular matrix remodeling in the form of adipose tissue expansion and fibrosis. Immune cell infiltration and chronic inflammation further contribute to tissue stiffening and adipose hypertrophy, highlighting the role of immune-mediated mechanisms in disease progression. The interplay between vascular, lymphatic, connective tissue, and immune dysfunction emerges as a central determinant of lipedema pathophysiology. Understanding these interconnected mechanisms is critical for elucidating the fundamental biology of lipedema, identifying novel biomarkers, and guiding the development of translational interventions and optimized clinical management strategies.

## 1 Introduction

Lipedema is a chronic and often debilitating adipose tissue disorder marked by disproportionate, symmetrical fat accumulation in the lower body, which may also affect the arms and other regions, resulting in mobility limitations and reduced quality of life (Al-Ghadban et al., 2025; Forner-Cordero et al., 2012; Brunner, 1982). Clinically, lipedema is characterized by easy bruising, tenderness to pressure, and a distinctive “cuffing” at the ankles—where swelling stops abruptly above the feet—helping distinguish it from lymphedema (Duhon et al., 2022; Herbst, 2012; Herbst et al., 2021). Often underdiagnosed, lipedema is commonly mistaken for obesity or lymphedema, leading to delayed diagnosis. In fact, national surveys in the United States and United Kingdom indicate an average diagnostic delay of 22 years after symptom onset (Fetzer and Fetzer, 2016). Progressive tissue heaviness, pain, and skin deformities can significantly impair mobility and quality of life (Al-Ghadban et al., 2025; Buso et al., 2019; Allen et al., 2020; Poojari et al., 2022; Torre et al., 2018; Aday et al., 2023; Aksoy et al., 2021). Epidemiological studies estimate that lipedema affects approximately 10%–11% of women worldwide (Buso et al., 2019; Kruppa et al., 2020), though prevalence is likely underestimated due to limited awareness among the public and healthcare providers. Additionally, the lack of a definitive diagnostic test has made the true incidence of lipedema difficult to pinpoint.

The pathophysiology of lipedema is complex and is primarily considered a disorder of loose connective tissue, characterized by fibrosis and extracellular matrix (ECM) dysregulation. Other features of the disease include the presence of genetic predispositions (Michelini et al., 2022; Michelini et al., 2020), hormonal alterations (Ka et al., 2023; Annunziata et al., 2024; Pinto da Costa Viana et al., 2025), chronic inflammation (Kruppa et al., 2023; Al-Ghadban et al., 2019; Grewal et al., 2025) and vascular dysfunction (Allerton, 2025; Strohmeier et al., 2022; Michelini et al., 2025). Among these factors, vascular dysfunction is increasingly recognized as a central contributor, as impaired microvascular integrity and increased capillary permeability promote local hypoxia, edema, and inflammation, leading to adipose tissue expansion and remodeling characteristic of lipedema. Detecting vascular dysfunction and elucidating its contribution to lipedema pathophysiology may be pivotal in distinguishing lipedema from obesity or lymphedema, as well as in identifying novel therapeutic targets. Histological studies have revealed dilated and leaky blood vessels, increased interstitial fluid accumulation, and fibrosis in lipedema tissue, consistent with vascular dysfuntion (Poojari et al., 2022; Al-Ghadban et al., 2019; Felmerer et al., 2020; Suga et al., 2009; Wolf et al., 2022; Bailey et al., 2026). Notably, while lymphatic vessels may remain structurally intact, their functionality appears compromised, potentially due to the overwhelming burden of fluid and waste products (Rasmussen et al., 2022; Duhon et al., 2022). This interplay between vascular, lymphatic, connective tissue, and immune dysfunction emerges as a central determinant of lipedema pathophysiology.

In this review, we aim to summarize the current evidence for vascular mechanisms underlying lipedema, and to better characterize vascular system involvement and its impact on patient care. Understanding these interconnected mechanisms is critical for elucidating the fundamental biology of lipedema, identifying novel biomarkers, and guiding the development of translational interventions and optimized clinical treatment strategies for Lipedema.

## 2 Endothelial dysfunction and vascular remodeling: Angiogenesis, fibrosis, and ECM changes

The endothelium, a layer of cells lining the entire vascular and lymphatic systems (Luscher and Vanhoutte, 1988; Boulanger, 2016) has been recently investigated in relation to lipedema development. Capillary vessels, composed of a single layer of endothelial cells (ECs), supported externally by a basement membrane (Hu et al., 2012; Germain et al., 2000) form semi-permeable barriers that regulate the transport of oxygen and carbon dioxide (Poole and Musch, 2023), ions (Lin and Sunner, 1994), nutrients (Poole and Musch, 2023), and water (Adiele et al., 2025; Martinović et al., 2025) between blood and surrounding tissue (Scallan et al., 2010; Murrant and Fletcher, 2022). During inflammation, vascular permeability increases, leading to fluid extravasation into the interstitial space (Weinbaum et al., 2021). The revised Starling principle highlights a prominent role for the endothelial glycocalyx in regulating this fluid exchange (Woodcock and Woodcock, 2012; Levick and Michel, 2010) and the resulting expansion of interstitial fluid volume, which adds to lymphatic load (Wiig and Swartz, 2012). Moreover, capillaries are highly dense throughout all tissues (A and ird, 2012; Becker et al., 2023), including adipose tissue depots, the primary tissues affected by lipedema (Guo et al., 2024; Dunaway et al., 2024; Bruno and Cilluffo, 2025).

### 2.1 Endothelial dysfunction in lipedema

In lipedema, endothelial function is impaired, as evidenced by disrupted junctions and increased vascular permeability (Strohmeier et al., 2022; Michelini et al., 2025). Primary human endothelial cells exposed to conditioned media from stromal vascular fraction (SVF) of lipedema patients show reduced expression of adherens junction protein VE-cadherin (CDH5) and tight junction protein ZO-1 (TJP1), while TIE2 (TEK receptor) expression remains unchanged (Strohmeier et al., 2022). TIE2 regulates migration and proliferation through its interaction with its ligand Angiopoietin-1 (Ang1) and normally stabilizes endothelial junctions by recruiting VE-cadherin and remodeling the cytoskeleton to maintain vascular integrity (Mitsuhashi et al., 2003). Activation of TIE2 promotes its localization to endothelial junctions, reinforcing barrier function and preventing leakage (Parikh, 2017; Zhang et al., 2024). In a lipedema, decreased vascular integrity leads to fluid accumulation within adipose tissue, which is recognized as a key feature of this disease (Strohmeier et al., 2022). Tissue studies report similar endothelial dysfunction, including decreased TIE2 expression, highlighting differences between *in vitro* and tissue-based conditions. Together, these findings demonstrate that impaired vascular integrity, secondary to dysfunction, underlies edema, pain, and easy bruising, which are characteristic features of lipedema (Buso et al., 2019).

**FIGURE 1:**
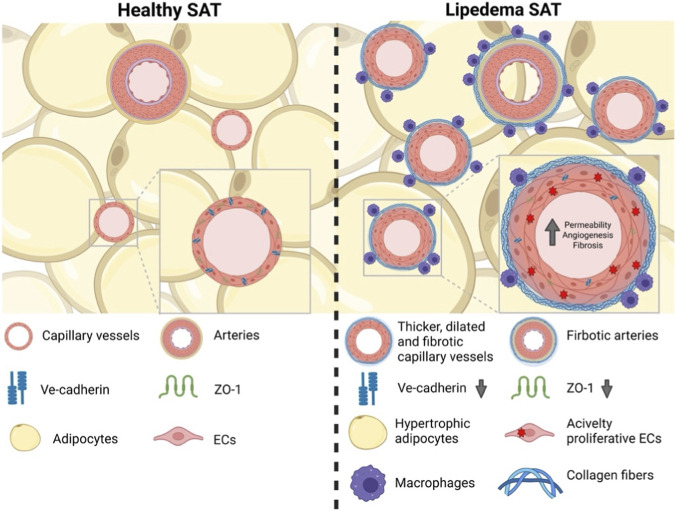
Thickened, dilated, and fibrotic vessels in lipedema-affected subcutaneous adipose tissue (SAT). Compared with healthy SAT, lipedema SAT exhibits increased endothelial permeability, enhanced angiogenesis, and perivascular fibrosis, with blood vessels surrounded by macrophages.

In addition to alterations in vascular permeability, other pathological features of endothelial dysfunction have been characterized in lipedema. Adipose tissue biopsies from female patients with stage 1 or 2 lipedema, healthy controls, and patients with obesity but without lipedema were analyzed to identify endothelial and vascular features specific to lipedema. This analysis revealed irregularities and thickening of the capillary vessels unique to lipedema-affected tissues. Moreover, these abnormal vessles were associated with an increased accumulation of M2 macrophages, which has recently been suggested as a feature specific to lipedema (Michelini et al., 2025; Wolf et al., 2022). The exact nature of what “thickening” means is yet to be determined. Taken together, these findings indicate a unique type of vascular phenotype in lipedema characterized by decreased endothelial integrity, macrophage infiltration, vessel fibrosis and thickening, and adipose hypertrophy (Figure 1).

### 2.2 Angiogenesis and endothelial proliferation

The adipose tissue endothelium in lipedema exhibits a hyperproliferative phenotype driving excessive angiogenesis (Michelini et al., 2025). Throughout the body, the process of angiogenesis is tightly regulated through signaling pathways which are activated in response to hypoxia, wound repair, inflammation and other stimuli (Flo et al., 2022). Some of the most canonical pathways regulating angiogenesis include the binding of TGF-β (transforming growth factor beta) to TGF-R (transforming growth factor beta receptor) (Zheng et al., 2001; van Meeteren et al., 2011), VEGF-A/C/D (vascular endothelial growth factor A/C/D) binding to VEGFR1/2 (vascular endothelial growth factor receptor 1/2) (Cao, 2009; Rahimi, 2006; Shibuya, 2006) or ANG1/2 binding to TIE2 (DeBusk et al., 2004; La Porta et al., 2018). Interestingly, emerging studies in the field of lipedema point to changes in the regulation of endothelial angiogenesis in the adipose tissue of patients (Al-Ghadban et al., 2019; Michelini et al., 2025; Siems et al., 2005). VEGF-A levels are twice as high in women with lipedema, appear unaffected by complex physical decongestive therapy (CPDT), despite its reported anti-fibrosclerotic effects. CPDT-comprising manual lymphatic drainage, compression, and/or shock wave therapy, has been studied in women with lipedema as well as in women with cellulitic edema unrelated to lipedema. Angiogenesis correlates with disease progression, with advanced stages showing elongated dermal vessels and lumens, consistent with increased permeability (Allen et al., 2020; Siems et al., 2005). Additionally, blood and lymphatic vessels in patients with lower extremity lipedema are characterized by increased capillary number and diameter, independent of BMI (Al-Ghadban et al., 2019). Compared to healthy controls and independent of BMI, lipedema patients had increased capillary number and diameter within the deep and superficial adipose compartments of the lower extremity (Al-Ghadban et al., 2019). Analyses using smooth muscle actin immunohistochemistry revealed higher microvessel density in the thigh skin of both non-obese and obese lipedema patients compared to controls. Increased capillary diameter was also observed in non-obese lipedema patients, providing evidence for angiogenic changes specific to lipedema rather than obesity. The origin of these robust small vessels networks observed in lipedema patients appears related to increased EC or pericyte proliferation, a finding not observed in obese patients (Michelini et al., 2025). What remains unclear is why the signaling pathways driving angiogenesis are upreagulated within specific areas of adipose tissue in patients with lipedema.

### 2.3 Fibrosis and extracellular matrix remodeling

Fibrosis is characterized by excessive deposition of ECM components, including collagen and proteoglycans (Henderson et al., 2020). It is well known that vascular changes are associated with tissue fibrosis. For example, systemic sclerosis (Varga and Abraham, 2007) involves a progressive vasculopathy associated with endothelial dysfunction, inflammation and impaired angiogenesis leading collagen deposition and tissue stiffening. Recent research has been described similar, but more focal, fibrotic changes in lipedema affected adipose tissue. In these studies, lipedema-affected thigh subcutaneous adipose tissue (SAT) was compared to non-affected abdominal SAT from patients with stage 1–3 lipedema. Using picrosirius red staining, fibrosis was greater in the lipedema-affected SAT compared to non—affected SAT and compared to non-lipedema control patients. Moreover, during lipedema progression there was decreased fibrosis in non-affected SAT, however in lipedema-affected SAT areas, fibrosis was elevated with each stage of disease (Kruppa et al., 2023). Despite increased overall fibrosis with disease progression, fibrotic changes were more localized in the hypervascular areas around blood vessels (Al-Ghadban et al., 2019; Curri and Merlen, 1986), reinforcing a close link between vascular instability and ECM deposition.

While it has become clear that there are distinct differences between lipedema and other conditions such as lymphedema (Duhon et al., 2022; Mahler et al., 2025), and obesity (Michelini et al., 2025; Greene and Sudduth, 2021), there has simultaneously been a growing appreciation of the interplay and overlap between lipedema and connective tissue disorders such as Ehlers-Danlos Syndrome (EDS) (Fiengo and Sbarbati, 2025; Wang et al., 2025). EDS arises from genetic defects in collagen structure that compromise ECM organization and fascia biomechanics, leading to joint hypermobility and vascular fragility. In patients with concomitant EDS and lipedema, both superficial and deep fascia are significantly thickened compared to individuals with EDS alone, indicating aberrant ECM deposition within adipose-associated connective tissue. Mechanistically, dysregulated expression of fibrillar collagens (COL1A1 and COL3A1), together with reduced MMP9 activity, may promote excessive perivascular collagen accumulation, impair endothelial–perivascular interactions, and destabilize the microvasculature (Poojari et al., 2022; Streubel et al., 2024). These alterations are consistent with disrupted angiogenic signaling, increased capillary fragility, and plasma leakage, establishing a permissive environment for localized fibrosis. These observations highlight the important role that ECM deposition and remodeling play in conjunction with endothelial dysfunction and fibrosis observed in lipedema and other vasculopathies and connective tissue disorders.

### 2.4 Interstitial fluid accumulation in endothelial dysfunction and vascular remodeling

**FIGURE 2:**
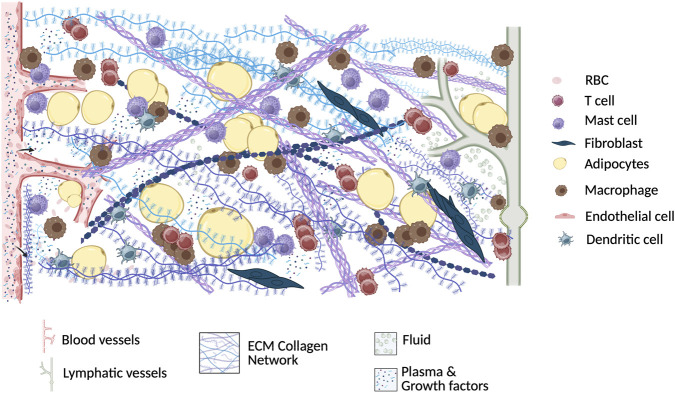
Effects of interstitial fluid accumulation on ECM remodeling, fibrosis, and inflammation. The schematic illustrates immune cell infiltration (macrophages, mast cells, and T cells) and microvascular fragility with endothelial dysfunction, leading to plasma leakage, interstitial fluid accumulation, and tissue edema. ECM, extracellular matrix; RBC, red blood cells.

Although lipedema and lymphedema are distinct disorders, they share the common feature of soft tissue swelling. In lymphedema, this swelling primarily results from impaired lymphatic clearance, whereas in lipedema, increased microvascular permeability and/or elevated vessel density, together with compromised lymphatic function, contribute to interstitial fluid accumulation. Fragile capillaries and endothelial dysfunction promote fluid leakage into the interstitial space, resulting in localized edema, tissue hypoxia, and non-pitting swelling in early disease stages (Herbst, 2012; Al-Ghadban et al., 2019; Faria et al., 2026). Persistent interstitial fluid imposes a sustained burden on the lymphatic system; although l ymphatic vessels in lipedema may initially compensate through increased pumping, chronic fluid overload eventually exceeds transport capacity, leading to lymphatic congestion and sustained tissue edema. Whereas in lymphedema primary lymphatic insufficiency or complete bloackage accelerates fluid accumulation from disease onset. Chronic edema alters tissue pressure and the local microenvironment, driving adipocyte hypertrophy and hyperplasia, fibroblast activation, and extracellular matrix remodeling, including fibrosis and altered collagen organization. These changes contribute to disproportionate expansion of subcutaneous adipose tissue, increased tissue stiffness, and chronic low-grade inflammation with immune cell infiltration (Figure 2). This inflammatory milieu further disrupts vascular and lymphatic function through cytokine-mediated increases in permeability and impaired lymphatic contractility, reinforcing interstitial fluid accumulation. Endothelial–adipocyte crosstalk likely contributes to pathological adipose expansion by influencing adipogenesis, lipid storage, and tissue remodeling, highlighting lipedema as a complex microvascular disorder characterized by progressive endothelial dysfunction, impaired fluid homeostasis, and maladaptive tissue remodeling (Kruppa et al., 2023; Al-Ghadban et al., 2019; Wolf et al., 2022; Rasmussen et al., 2022).

## 3 Inflammation in lipedema: metabolic and immune drivers

Lipedema is a progressive disease characterized by adipose hypertrophy, tissue pain, immune cell recruitment, and fibrosis. Although historically underdiagnosed and frequently misclassified as obesity or lymphedema, increasing clinical, histological, and molecular evidence supports the concept that lipedema is driven by persistent chronic inflammation, immune dysregulation, and pathological tissue remodeling. Unlike obesity-associated inflammation, which is often related to insulin resistance and metabolic disruption, lipedema inflammation appears disproportionate to body mass index and localized predominantly to affected subcutaneous fat depots (Al-Ghadban et al., 2019).This low-grade inflammatory environment likely contributes to hallmark clinical features of lipedema, including pain on palpation, tenderness, easy bruising, and progressive tissue fibrosis. Notably, inflammation in lipedema does not resolve but instead appears to be self-sustaining, suggesting impaired immune resolution mechanisms within affected adipose tissue (Faria et al., 2026; Kruppa et al., 2026; Atabilen Pinar et al., 2025).

### 3.1 Macrophage infiltration and immune imbalance

Among immune populations, macrophages represent a central driver of lipedema-associated inflammation. Multiple studies report increased macrophage density in lipedema adipose tissue compared with BMI-matched controls (Poojari et al., 2022; Al-Ghadban et al., 2019; Wolf et al., 2022; Grewal et al., 2025). However, macrophage polarization in lipedema is distinct from the classical pro-inflammatory M1-predominant profile observed in obesity. Although macrophages in lipedema adipose tissue show an M2-like (CD163^+^) signature, the tissue exhibits a persistent cytokine/growth-factor milieu and progressive remodeling (e.g., fibrosis and vascular/lymphatic signaling changes), suggesting that macrophage programs are skewed toward immunoregulatory/pro-remodeling functions rather than effective resolution of inflammation (Kruppa et al., 2023; Grewal et al., 2025; Felmerer et al., 2020; Wolf et al., 2022). Crosstalk between classically activated M1 and dysfunctional M2-macrophages generates a hybrid inflammatory phenotype that sustains tissue remodeling, angiogenesis, and fibrosis rather than restoring homeostasis.

### 3.2 Cytokine-driven angiogenesis and fibrosis

Consistent with the imbalanced M1/M2 macrophages activity, inflammatory signaling is elevated in lipedema adipose tissues. Several studies have reported increased Interleukin-1 beta (IL-1β) expression, implicating innate immune activation and vascular dysfunction that disrupts endothelial barrier integrity (Kruppa et al., 2023; Felmerer et al., 2020). Beyond its inflammatory effects, IL-1β is a well-recognized mediator of nociceptor sensitization linking local inflammation to the chronic pain reported in lipedema (Al-Ghadban et al., 2019).

In parallel, VEGF-C is consistently elevated in lipedema adipose tissue (Al-Ghadban et al., 2019; Felmerer et al., 2020; Fel et al., 2020). While VEGF is essential for angiogenesis, its chronic elevation promotes the formation of immature, structurally abnormal, and hyperpermeable blood vessels. IL-1β and VEGF pathways are tightly interconnected: IL-1β promotes lymphangiogenic signaling by inducing VEGF-C/VEGF-D production, from macrophages, inflamed endothelial cells, and fibroblasts, thereby amplifies inflammatory signaling and vascular dysfunction (Felmerer et al., 2020; Kruglikov and Scherer, 2024; Baluk et al., 2013). This reciprocal reinforcement establishes a microenvironment characterized by chronic inflammation, pathological angiogenesis, and increased vascular permeability (Cao et al., 2004; Tacconi et al., 2015; Breslin et al., 2007).

A defining feature of lipedema is excess, fragile vasculature with increased vascular permeability driven in part by chronic inflammation (Kruppa et al., 2020; Michelini et al., 2025; Szolnoky et al., 2017). Pro-inflammatory cytokines such as IL-1β and TNF-α disrupt endothelial junctional integrity (VE-cadherin) and increase vascular leakness, while VEGF-C mediated signaling promotes pathological vascular and lymphatic remodeling characterized by impaired barrier function, abnormal vessel structure, and increased permeability under chronic inflammatory conditions (Xiong et al., 2020; Hofmann et al., 2002). Consequently, lipedema tissue exhibits leaky capillaries, interstitial edema, and increased susceptibility to bruising. Accumulation of interstitial fluid further impairs oxygen diffusion, creating a hypoxic microenvironment that stimulates additional angiogenic and inflammatory signaling, thereby reinforcing this pathological cycle (Duhon et al., 2022; Jiang et al., 2022). Fibroblasts adopt an activated, profibrotic phenotype, leading to excessive extracellular matrix deposition and progressive fibrosis (Kruppa et al., 2023; Grewal et al., 2025). This remodeling increases tissue stiffness, impairs lymphatic drainage, and exacerbates local hypoxia, further amplifying macrophage-driven inflammation and cytokine–angiogenic signaling.

### 3.3 Succinate and SUCNR1 axis in lipedema

Multiple features of lipedema including fibrotic expansion of subcutaneous adipose tissue, edematous adipose tissue, and chronic low-grade inflammation, all contribute to a hypoxic adipose microenvironment. Under hypoxic and inflammatory stress, disruption of mitochondrial oxidative metabolism leads to succinate accumulation, which functions as a key metabolic signal (Selak et al., 2005; Wojtovich et al., 2013). Elevated succinate inhibits prolyl hydroxylase (PHD) activity, thereby stabilizing hypoxia inducible factor 1 subunit alpha (HIF-1α, a master transcriptional regulator of hypoxic and inflammatory responses (Tannahill et al., 2013; Palsson-McDermott and O'Neill, 2025). In parallel, hypoxia-associated mitochondrial dysfunction and succinate oxidation through reverse electron transport increase reactive oxygen species (ROS) production, which further enhances HIF-1α stabilization and activates redox-sensitive inflammatory pathways. Together, succinate-driven HIF-1α signaling and ROS generation establish a feed-forward loop that sustains inflammatory gene expression under conditions of metabolic stress.

Beyond its intracellular effects, succinate also acts as an extracellular ligand that activates succinate receptor-1 (SUCNR1) in mammalian cells. Succinate signaling has been shown to promote IL-1β production, macrophage activation, and angiogenesis across multiple disease contexts (Mills and O'Neill, 2014; Littlewood-Evans et al., 2016; Guo et al., 2022). SUCNR1 is expressed on macrophages, endothelial cells, fibroblasts, and adipose cells, positioning it as a central integrator of metabolic and inflammatory cues. Activation of SUCNR1 on macrophages promotes IL-1β secretion and inflammatory gene expression, while SUCNR1 signaling in endothelial cells promotes VEGF production, angiogenesis, and increased vascular permeability (Littlewood-Evans et al., 2016; Guo et al., 2017).

In the context of lipedema, aberrant succinate–SUCNR1 signaling could simultaneously drive macrophage dysfunction, amplify IL-1β and VEGF expression, and exacerbate vascular leakiness. In macrophages, activation of SUCNR1 has been shown to promote classical M1-like polarization, characterized by glycolytic metabolism and increased production of pro-inflammatory cytokines, such as IL-1β, TNF-α, and MCP-1 (van Diepen et al., 2017). Under certain pathological conditions, succinate–SUCNR1 signaling can skew polarization toward a hyperactivated M2-like phenotype, facilitating angiogenesis, lymphangiogenic, and fibrotic remodeling within adipose tissue (Al-Ghadban et al., 2019; Wolf et al., 2022; Pu et al., 2024; Trauelsen et al., 2021; Ran and Montgomery, 2012). Succinate–SUCNR1 signaling is well characterized in vascular endothelial cells, where it regulates pro-angiogenic programs, endothelial inflammatory signaling, and metabolic pathways (Sapieha et al., 2008; Atal et al., 2021; Toma et al., 2008; Atallah et al., 2024). In contrast, its role in lymphatic vessels and lymphangiogenesis remains largely unexplored, especially in inflammatory or hypoxic settings. Elevated succinate activates SUCNR1 can influence immune cell trafficking through lymphatic pathways. Succinate-SUCNR1 signaling is known to promote chemotaxis of dendritic cells (DCs) toward lymph nodes and shape local inflammatory responses in both human and mouse models (Rubic et al., 2008; Saraiva et al., 2018). In murine models of rheumatoid arthritis, SUCNR1 functions as a chemotactic gradient sensor that recruits DCs from peripheral tissues into the draining lymph nodes, while deletion of SUCNR1 significantly reduces DCs infiltration and downstream T-helper-17 expansion in lymphoid organs. DCs entry into lymphatic vessels and migration to lymph nodes is a key step in immune surveillance. Dysregulated SUCNR1-dependent chemotaxis may plausibly contribute to perturbed lymphatic overload and immune persistence in lipedema, particularly as tissue shifts towards chronic inflammation, and disease progresses toward late-stage lipolymphedema. Collectively, these observations position SUCNR1 as a compelling mechanistic link between metabolic stress, chronic inflammation, and vascular pathology in lipedema.

### 3.4 Targeting SUCNR1 as therapeutic approach for lipedema

**FIGURE 3:**
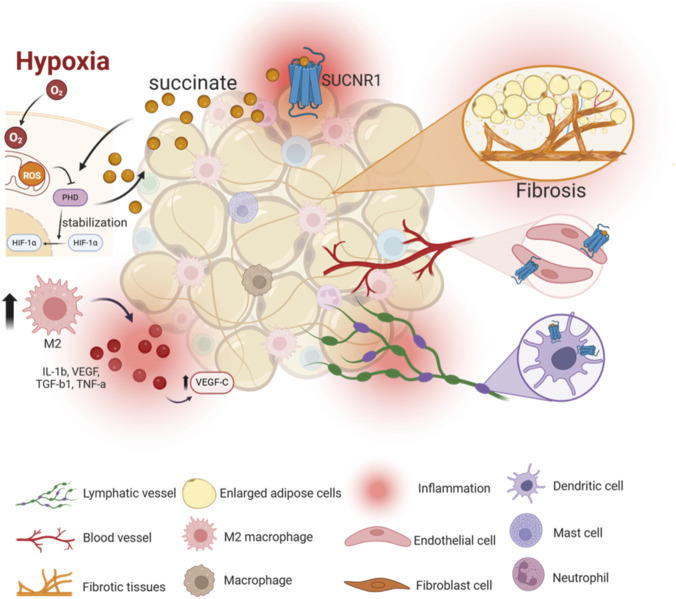
Hypoxia and chronic inflammation induced succinate accumulation in lipedema activates SUCNR1 on adipose cells, macrophages, endothelial cells, and dendritic cells. SUCNR1 signaling orchestrates macrophage polarization imbalance, IL-1β and VEGF-C upregulation, and dendritic cells infiltration into lymphatic vessels. Sustained SUCNR1-mediated immune-vascular-lymphatic dysregulation reinforces edema, fibrosis, and adipose tissue remodling.

Targeting inflammatory signaling pathways has long been proposed as a strategy for lipedema management, yet effective disease-modifying therapies remain lacking. The identification of SUCNR1 as a metabolic–immune sensor raises the possibility of selective therapeutic intervention that could dampen inflammation while preserving essential immune functions. Preclinical studies across multiple inflammatory and vascular disease models demonstrate that pharmacological inhibition or genetic ablation of SUCNR1 attenuates inflammatory and angiogenic responses. In these settings, blockade of SUCNR1 signaling reduces the production of key pro-inflammatory cytokines, including IL-1β and TNF-α, and dampens downstream activation of inflammatory transcriptional programs (Figure 3). In parallel, SUCNR1 inhibition limits pathological angiogenesis, reduces aberrant VEGF signaling, and alleviates tissue edema and vascular dysfunction (Mu et al., 2017; Klid et al., 2025; Atallah et al., 2022).

Given its central role at the intersection of metabolism, immunity, and vascular biology, SUCNR1 represents a promising candidate for future translational studies in lipedema. Modulation of SUCNR1 signaling may complement hormone-based or vascular-targeted therapies by addressing the upstream inflammatory drivers of disease progression.

## 4 Biomedical imaging of vasculature and inflammation in lipedema

### 4.1 Venous imaging

Vascular abnormalities involving the arterial, venous, and lymphatic systems in lipedema have been documented by multiple biomedical imaging modalities. These findings are largely consistent with clinical presentation of the disease and provide important insights into its underlying pathophysiology. Chronic venous insufficiency (CVI) is a condition of insufficient peripheral venous blood circulation detected by Doppler ultrasonography. New evidence suggests CVI is among the most prevalent comorbidities of lipedema. In a retrospective study of 381 females with lipedema in a hospital in Switzerland, 86.2% were diagnosed with CVI, making it more prevalent than obesity as a comorbidity (Luta et al., 2025). Similarly, in a vascular medicine clinic in the United States, CVI was reported in 50% of the cases with lipedema (Dean et al., 2020). It remains unknown whether symptoms of CVI relate to other blood vascular symptoms, such as easy bruising at the capillary level. Further research is warranted to assess both macro and microvascular venous characteristics of lipedema by clinical assessment, biomedical imaging, and histology. In addition to venous involvement, patients may meet clinical criteria for lymphedema when edema is unresolved with elevation, in accordance with International Society of Lymphology (ISL) guidlines (Herbst, 2012; Tiwari et al., 2003). When diagnostic criteria for both conditions are fulfilled, the phenotype is commonly referred to as “lipolymphedema” or lipedema with secondary lymphedema (Torre et al., 2018; Dean et al., 2020; Radhakrishnan and Rockson, 2008).

### 4.2 Arterial imaging

Evidence for arterial system involvement in lipedema has also begun to emerge. Neuroimaging by noninvasive arterial spin labeling MRI measured elevated arterial perfusion by cerebral arterial blood flow quantification in patients with lipedema compared to BMI-matched controls (57.3 ± 9.6 vs. 49.8 ± 8.3 mL/100 g/min, p = 0.02) (Petersen et al., 2020). Neuroimaging of anatomy and vascular morphology did not observe any evidence of large vessel vasculopathy in the brain of patients with lipedema in this cohort. This study motivated an observational clinical trial to observe arterial, venous, and lymphatic systems by MRI in patients with lipedema compared to healthy controls which is ongoing (https://clinicaltrials.gov/study/NCT05464927).

### 4.3 Lymphatic imaging

Imaging of the lymphatic system in lipedema has become an active area of research. Lymphoscintigraphy is the gold-standard technique for observing lymphatic transport dysfunction in lymphedema and has been applied to lipedema. Lymphoscintigraphy demonstrates delayed transport function, distal collateral flow, dilated or tortuous vessels, and additional lymphatic vessels in certain cohorts with lipedema (Ceylan and Yilmaz, 2023; Chachaj et al., 2023; Tartaglione et al., 2020; van de Pas et al., 2020). However, findings across studies remain inconsistent, as summarized in a comprehensive review by van der Parra and colleagues (van la Parra et al., 2024). More recently, optical imaging using indocyanine green near infrared (ICG-NIR) lymphography has demonstrated an increased number of lymphatic vessels in lipedema (Zaleska et al., 2023). Additionally, lymphatic vessels appear to be pumping faster than normal in two independent cohorts (Rasmussen et al., 2022; Zaleska et al., 2023), suggesting a potential compensatory response to increased lymphatic load. Lymphoscintigraphy and ICG-lymphography rely on dermal injection of an external substance and tracing it through tissue, called “tracers”. Tracer-based lymphatic imaging only probes the territory accessed by the injection. As a result, deeper lymphatic and adipose tissue compartments may not be adequately captured by these modalities. Thus, imaging modalities which resolve vasculature in deeper adipose tissue such as magnetic resonance imaging (MRI), are being explored in lipedema.

Using MRI, noninvasive imaging of lymphatic collectors and tissue edema can be achieved by heavily T2-weighted MR lymphangiography (MRL) (Crescenzi et al., 2023; Cellina et al., 2020). MRL displays hyperintense signal patterns that correspond to edema in lymphedema, where the source of lymphatic insufficiency is known (Crescenzi et al., 2017). Hyperintensities on MRL are also observed in lipedema, to a greater extent than in BMI-matched female controls without lipedema (Crescenzi et al., 2023). Hyperintensities on MRL are more diffuse in lipedema adipose tissue than in cancer-related lymphedema. Another study corroborated hyperintensities on MRL in patients with “lipolymphedema” (Cellina et al., 2020). These observations were interpreted as a full lymph load in affected tissues of patients with lipedema that exceed transport capacity. Adipose tissue edema observed on MRL is consistent with clinical symptoms of non-pitting edema and tissue heaviness in lipedema with lymphedema (Duhon et al., 2022; Aday et al., 2023; Crescenzi et al., 2023).

### 4.4 Tissue sodium imaging as an indicator of vascular disease

**FIGURE 4:**
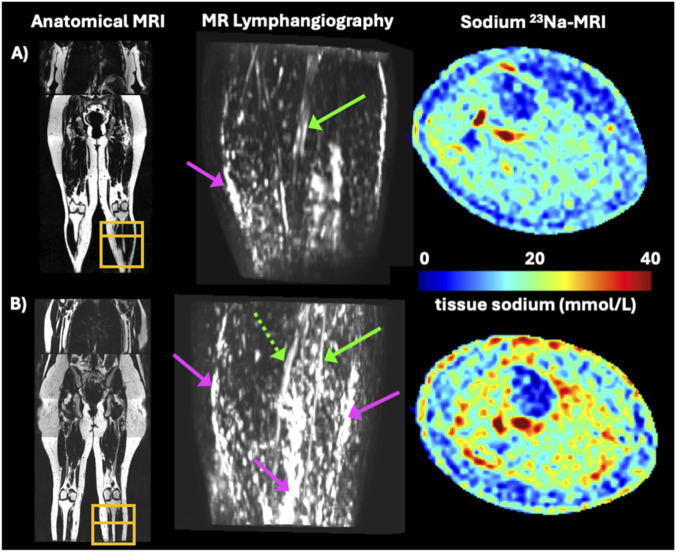
Biomedical imaging by MRI in a case with **(A)** lipedema (age = 46 years, BMI = 24.9 kg/m^2^) compared to **(B)** obesity (age = 45 years, BMI = 38.5 kg/m^2^). MR lymphangiography shows features of vascular involvement (green arrows: dilated vessels, dotted arrow: tortuous vessel, pink arrow: tissue edema) to a greater extent in lipedema. Sodium 23Na MRI reveals higher leg tissue sodium in the case of lipedema. Dilated vasculature with excess tissue sodium is interpreted as an inflammatory profile in lipedema which can be imaged *in vivo* with MRI.

Molecular imaging of lipedema has revealed tissue composition of the affected legs that is consistent with vascular imaging and histological findings. Initial studies sought to measure the subcutaneous adipose tissue volume in legs with lipedema. By MRI, subcutaneous adipose tissue is 42% larger in the calf of people with lipedema than those without lipedema with similar body-mass-index (BMI) (p < 0.001) (Crescenzi et al., 2020; Crescenzi et al., 2018). By Dual-Energy X-ray Absorptiometry (DEXA), the leg fat mass normalized by total body fat mass was 27% higher in lipedema than controls (p < 0.001) (Buso et al., 2022). Beyond adipose tissue volume, the characteristics of adipose tissue in lipedema have been characterized *in vivo* by sodium ^23^Na MRI. Sodium MRI is a noninvasive molecular imaging technique which measures standardized tissue sodium content in biological tissue. In lipedema, tissue sodium content was significantly higher in legs with lipedema compared to controls, despite being matched for BMI (Crescenzi et al., 2018). Tissue sodium was also elevated in the legs compared to arms of patients with lipedema, indicating local sodium retention in areas most affected by lipedema. Tissue sodium retention has been discovered in a host of other cardiovascular diseases and is thought to indicate poor circulation and resulting inflammation (Gast et al., 2023). Sodium retention can also be found in tissues with fibrosis or diseased cartilage and is therefore interesting to observe in lipedema adipose tissue where histological fibrosis is observed (Figure 4).

Collectively, biomedical imaging studies support the presence of alterations across all three vascular systems—venous, lymphatic, and arterial—in lipedema. Based on current evidence, a working hypothesis is that lymphatic load exceeds transport capacity in lipedema following a convergence of inflammation, adipocyte hypertrophy, and arterial angiogenesis and venous insufficiency (Duhon et al., 2022). The extent to which venous and lymphatic dysfunction interact, and whether these abnormalities define disease subtypes or correlate with disease severity and progression, remains to be determined. Emerging multimodal imaging approaches, such as combined ultrasound and photoacoustic imaging, may enable simultaneous assessment of blood and lymphatic vasculature and help address these questions (Park et al., 2025; Wang et al., 2020). Further mechanistic studies could combine imaging modalities with biological assays to determine how tissue pathology relates to vascular function in lipedema. By using biomedical imaging, translational methods could be developed into diagnostic and therapeutic biomarkers of lipedema.

## 5 Discussion

Lipedema is a chronic inflammatory microvascular disease marked by adipose tissue hypertrophy, immune activation, vascular remodeling, endothelial dysfunction, tissue fibrosis, and impaired fluid homeostasis (Al-Ghadban et al., 2019; Strohmeier et al., 2022; Michelini et al., 2025; Felmerer et al., 2020; Rabiee, 2025). In this review, we highlight that, unlike obesity-related inflammation, driven by systemic metabolic dysfunction, lipedema inflammation is localized, persistent, and disproportionate to body mass index, suggesting disease-specific regulatory failures within affected subcutaneous adipose tissue (Jeziorek et al., 2025; Vazirnia et al., 2026). This localized inflammatory milieu initiates a cascade of cellular and vascular dysfunctions that together drive the hallmark clinical features of lipedema.

Macrophage accumulation is a consistent feature of lipedema tissue; however, their polarization profile differs from classical obesity-associated M1 dominance. Instead, lipedema macrophages exhibit an M2-like or hybrid phenotype that promotes angiogenesis, fibrosis, and extracellular matrix remodeling rather than restoring tissue homeostasis (Kruppa et al., 2023). This dysfunctional macrophage state may explain how lipedema tissue sustains chronic inflammation without overt systemic inflammatory markers. The persistence of cytokines such as IL-1β, together with growth factors including VEGF, establishes a tissue microenvironment that favors vascular instability, nociceptor sensitization, and progressive fibrotic remodeling (Al-Ghadban et al., 2019; Fel et al., 2020; Ernst et al., 2023).

This inflammatory environment is further amplified by metabolic stress. Hypoxia resulting from adipocyte hypertrophy, edema, and fibrosis disrupts mitochondrial oxidative metabolism, leading to succinate accumulation. Succinate functions not merely as a metabolic intermediate but as a signaling molecule that stabilizes HIF-1α and activates SUCNR1 on immune, endothelial, and stromal cells (Huang et al., 2024; Keiran et al., 2019). This axis can synchronize inflammatory cytokine production, angiogenesis, and vascular permeability, placing succinate–SUCNR1 signaling at the nexus of metabolic and immune dysfunction in lipedema. Although direct evidence for SUCNR1 activation in lipedema tissue remains limited, the convergence of hypoxia, IL-1β, VEGF elevation, macrophage dysfunction, and angiogenesis strongly supports this pathway as a plausible driver of disease progression.

Notably, several limitations should be considered in interpreting current findings. Many lipedema studies are based on small sample sizes, reflecting challenges in patient recruitment and standardized tissue collection. In addition, heterogeneity in disease stage and variability in BMI matching across studies may contribute to inconsistent observations. Finally, while the succinate–SUCNR1 axis provides a compelling mechanistic framework, its activation in human lipedema tissue remains largely hypothetical and requires direct experimental validation.

Endothelial dysfunction emerges as a second unifying hallmark (Allerton, 2025; Michelini et al., 2025). Lipedema tissue displays increased capillary density, endothelial proliferation, impaired junctional integrity, and fragile vasculature. These features provide a mechanistic explanation for clinical symptoms such as easy bruising, pain, and edema (Herbst et al., 2021; Kruppa et al., 2026; Vazirnia et al., 2026). Importantly, endothelial abnormalities in lipedema differ from those observed in obesity, supporting the concept that lipedema is a distinct vascular–adipose disorder rather than a variant of metabolic disease. The association of hypervascular regions with localized fibrosis further suggests that endothelial dysfunction precedes and potentially drives fibrotic remodeling, as observed in other chronic inflammatory conditions.

The lymphatic system represents both a responder to and amplifier of these pathological processes. Imaging studies reveal increased lymphatic vessel number and pumping in early disease, consistent with a compensatory response to elevated interstitial fluid load driven by microvascular leakiness (Rasmussen et al., 2022; Zaleska et al., 2023; Crescenzi et al., 2017). Over time, however, sustained fluid overload exceeds lymphatic transport capacity, leading to tissue edema and secondary lymphatic dysfunction, particularly in advanced lipedema. This model contrasts with primary lymphedema, where lymphatic insufficiency is the initiating event, and highlights fundamental differences in disease trajectory despite overlapping clinical features.

Interstitial fluid accumulation thus serves as a central mediator linking immune activation, endothelial dysfunction, and tissue remodeling. Chronic edema alters tissue pressure, oxygenation, and cellular crosstalk, promoting adipocyte hypertrophy, fibroblast activation, and extracellular matrix deposition (Allen et al., 2020; Al-Ghadban et al., 2019). These changes further impair vascular and lymphatic function, reinforcing fluid retention and inflammation. The interstitium thus emerges not as a passive compartment but as an active signaling hub that integrates metabolic, immune, and vascular cues in lipedema.

Building on this concept of tissue-level compartmentalization, depot-specific adipose biology provides an additional layer of mechanistic specificity in lipedema. It is important to note that subcutaneous and visceral adipose tissues differ substantially in their intrinsic biological properties, including adipose-derived stem cell origin and characteristics, transcriptomic profiles, and hormonal responsiveness (e.g., to estrogen) (Pinto da Costa Viana et al., 2025; Streubel et al., 2024; Faria et al., 2026; Karastergiou et al., 2012; Tchkonia et al., 2013). Subcutaneous adipose tissue–derived stem cells exhibit distinct proliferative, inflammatory, and differentiation programs compared with those from visceral depots, which may influence tissue remodeling capacity, vascular behavior, and extracellular matrix dynamics. These depot-specific properties may therefore independently contribute to susceptibility to lipedema and its regional distribution (Al-Ghadban et al., 2019; Kruppa et al., 2026).

Extending this depot-level heterogeneity, a key unresolved question in lipedema pathophysiology is the striking anatomical selectivity of affected adipose depots (Al-Ghadban et al., 2019; Al-Ghadban et al., 2020). From a vascular perspective, two distinctions are particularly relevant. First, within subcutaneous adipose tissue, lipedema predominantly affects the lower extremities rather than abdominal or upper body depots (Al-Ghadban et al., 2025; Herbst et al., 2021), suggesting region-specific vascular or stromal susceptibility. Second, visceral adipose tissue is consistently spared, despite systemic metabolic and inflammatory signaling (Herbst, 2012; Vazirnia et al., 2026). Together, these observations indicate that lipedema is not solely driven by generalized adiposity or edema but reflects hierarchical depot- and region-specific vascular–stromal vulnerability, likely shaped by differences in vascular density, lymphatic architecture, mechanical load, and adipose stem cell–endothelial crosstalk (Strohmeier et al., 2022; Michelini et al., 2025; Fel et al., 2020). Incorporating this anatomical selectivity strengthens the concept of lipedema as a distinct vascular–adipose disorder rather than a uniform expansion of subcutaneous fat.

Finally, emerging links between lipedema and connective tissue disorders such as EDS underscore the importance of ECM integrity in disease pathophysiology (Wang et al., 2025). Collagen dysregulation, reduced matrix turnover, and altered fascia structure may contribute to vascular fragility, pain sensitivity, and perivascular fibrosis, offering a unifying explanation for shared clinical features such as bruising and tissue tenderness. Collectively, these observations further support the classification of lipedema as a disorder of adipose–vascular–connective tissue interplay rather than isolated fat accumulation.

## 6 Conclusion

The etiology of lipedema remains elusive. Emerging evidence supports a disease model in which adipocyte hypertrophy and microvascular instability interact to disrupt tissue homeostasis. Lipedema tissue pathology is characterized by endothelial dysfunction, aberrant angiogenesis, and extracellular matrix remodeling. These tissue changes promote chronic inflammation, interstitial fluid accumulation, and immune dysregulation. Metabolic stress and hypoxia within affected adipose depots may sustain inflammatory signaling through pathways such as succinate–SUCNR1, linking immune activation to vascular permeability and fibrosis. Biomedical imaging reveals angiogenesis, venous insufficiency, and tissue edema features consistent from the micro-to macro-scale. Continued research is needed to understand the vascular etiology of lipedema and identify biomarkers to translate discovery to evidence-based clinical care for patients with lipedema.
